# Identification of Prognostic Genes and Immune Landscape Signatures Based on Tumor Microenvironment in Lung Adenocarcinoma

**DOI:** 10.1155/2022/6703053

**Published:** 2022-08-18

**Authors:** Pengkai Han, Yunxiu Fan, Qiping Liu, Junhao Zhou

**Affiliations:** ^1^Department of Pulmonary and Critical Care Medicine, Chongqing University Three Gorges Hospital, Chongqing 404100, China; ^2^Department of Oncology, Chongqing University Three Gorges Hospital, Chongqing 404100, China

## Abstract

**Background:**

Lung adenocarcinoma is the most common lung cancer subtype and accounts for the highest proportion of cancer-related deaths. The tumor microenvironment influences prognostic outcomes in lung adenocarcinoma (LUAD).

**Materials and Methods:**

We used the ESTIMATE algorithm (Estimation of STromal and Immune cells in MAlignant Tumor tissues using Expression data) to investigate the role of microenvironment-related genes and stromal cells in lung adenocarcinoma prognosis. This analysis was done on lung adenocarcinoma cases from The Cancer Genome Atlas (TCGA). The cases were divided into high and low groups on the basis of immune and stromal scores, respectively.

**Results:**

There were close correlations between immune scores with prognosis and disease stage. There were 367 differentially expressed genes. Combining the Gene Expression Omnibus (GEO) database, we found 14 prognosis-related genes.

**Results:**

There were close correlations between immune scores with prognosis and disease stage. There were 367 differentially expressed genes. Combining the Gene Expression Omnibus (GEO) database, we found 14 prognosis-related genes. *Results*. Based on the enrichment levels of the immune cell types, we clustered LUAD into Immunity_H and Immunity_L subtypes. Most of these genes were upregulated in Immunity_H subtype. Finally, using the Human Protein Atlas (HPA) and the Clinical Proteomic Tumor Analysis Consortium (CPTAC) databases, most of the proteins corresponding to prognostic genes were verified to be differentially expressed between the tumor and normal groups.

**Conclusions:**

The key genes identified in this study are involved in molecular mechanisms of LUAD.

## 1. Introduction

Globally, lung cancer is the most common tumor type and the leading cause of tumor-related deaths. Non-small-cell lung cancer (NSCLC) accounts for more than 80% of all lung cancer cases. Lung adenocarcinoma (LUAD) is a common class of NSCLC [1]. LUAD constitutes half of all lung cancers and is associated with high morbidity rates.

Despite advances in screening, diagnosis, and lung cancer management, clinical outcomes remain poor. Lung tumors with different genetic and biological features may have different prognoses and drug responses. Genomic and biological features have been considered for prediction of cancer risks [2, 3]. However, tumorigenesis is driven by multiple factors and mechanisms. Tumor tissues comprise the parenchyma and interstitium. Tumor cells make up the parenchyma and are the main components of the tumor while the interstitium, which is composed of connective tissues, blood vessels, and immune cells and provides support and nourishment. Coevolution of tumor cells and the tumor microenvironment (TME) provides the basis for cancer cell proliferation and metastasis. Tumor cells have a remarkable ability for infinite proliferation, escape from the immune system, local invasion, and distant metastasis. Various factors in the TME promote cancer progression [4]. Stromal cells influence tumorigenesis and cancer progression [5] and drug fast [6]. Tumor cells and recruited immune cells in the tumor microenvironment contribute to cancer outcomes [7]. As such, assessing TME functions has been proposed as a diagnostic strategy [8]. Currently, the role of the TME, which is composed of immune and stromal cells, in cancer progression is vaguely understood. In LUAD, it has not been fully established how tumor biomechanisms and TME variations affect patient outcomes.

Innate tumor genes determine tumor occurrence and development. The TME influences gene expressions and cancer outcomes [9, 10]. The TME can be evaluated by methods based on mRNA expression data. The ESTIMATE (Estimation of STromal and Immune cells in MAlignant Tumor tissues using Expression data) algorithm was used to assess immune and stromal cell infiltrations [10]. Gene expression and clinical data were obtained from The Cancer Genome Atlas (TCGA) and Gene Expression Omnibus (GEO) database [11].

Using LUAD gene expression data from TCGA, ESTIMATE was applied to evaluate the infiltrations of cells constituting the TME and screened a series of TME-associated genes. Further, survival analysis of these genes was performed using TCGA and GEO databases. Finally, corresponding protein expressions of each prognostic gene were validated.

## 2. Materials and Methods

### 2.1. Data Acquisition and Screening

Patient information for LUAD gene expressions as of May 2020 was downloaded from TCGA database [11] (https://portal.gdc.cancer.gov/). We analyzed 594 samples, of which 59 were normal lung tissues while 535 were tumor tissues. Clinical data included gender, age, and prognosis. Gene IDs were annotated in the gene transfer format. For validation analysis, gene expression data for LUAD patients were downloaded from the GEO database (GSE3141, GSE30219, and GSE31210 datasets) (http://www.ncbi.nlm.nih.gov/geo) along with disease outcome data. The above cases with expression data were included in the study. For survival analysis, only cases with survival information were selected.

### 2.2. Evaluation of Immune Cells and Acquisition of ESTIMATE Scores

The ssGSEA procedure was used to obtain enrichment scores for each specific term. Marker genes for 29 immune cell subtypes were obtained from published literature (Table S1). The ESTIMATE score system consists of the immune and stromal scores [12]. ESTIMATE analyzes bulk tumor data and predicts tumor purity and immune as well as stromal cell infiltrations by single-sample gene set expression analysis (ssGSEA) [10]. Immune and stromal scores denote immune and stromal cell infiltrations, respectively. ESTIMATE analysis was performed using “ESTIMATE” in R (https://bioinformatics.mdanderson.org/estimate/rpackage.html). Patients were assigned into different groups depending on ESTIMATE scores. Survival time for patients in different groups was compared by survival analysis. Differences in ESTIMATE scores among clinical stages were also investigated.

### 2.3. Identification of Differentially Expressed Genes (DEGs)

The limma R package [13] for analysis of high-throughput genomic data was used to analyze the corresponding dataset. With the mean of immune and stromal scores as the boundary values, all cancer cases were assigned into low and high groups. The original *P* values were adjusted. False discovery rate (FDR) and fold changes (FC) were calculated to identify DEGs. The intersection of DEGs obtained by immune score and matrix scores was determined by Venn diagram analysis.

### 2.4. GO and KEGG Enrichment Analysis

GO term analysis was performed to elucidate on the biological processes (BP), cellular components (CC), and molecular functions (MF) in which the genes were enriched. GO and KEGG enrichment analyses were performed using clusterProfiler and DOSE packages in R [14]. *P* < 0.05 indicated significantly enriched pathways.

### 2.5. Overall Survival Analysis of DEGs

The relationship between survival time and gene expression levels of DEGs was determined by the log-rank test. Kaplan-Meier curves were plotted to visualize the relationships.

### 2.6. Clustering Analysis

Based on enrichment levels (ssGSEA scores) of the 29 immune cells, hierarchical clustering of LUAD was performed to identify different patterns of immune cell infiltrations and divided the LUAD cases into Immunity_H and Immunity_L subtypes. Then, differences in ESTIMATE scores and prognostic gene expressions between the immune subtypes were compared.

### 2.7. Assessment of Prognostic Gene Expressions at the Protein Level

Protein expressions of prognostic genes were validated using the Clinical Proteomic Tumor Analysis Consortium (CPTAC, https://proteomics.cancer.gov/programs/cptac) and the Human Protein Atlas databases (HPA, https://www.proteinatlas.org). Immunohistochemical and proteomic results were explored to verify their differential expressions in tumor and normal tissues. This study was conducted according to the flow chart shown in Figure S1.

### 2.8. Statistical Analysis

The R software (version 3.5.3; https://www.r-project.org/) was used for statistical analysis and to visualize the results. ESTIMATE package was used to run ESTIMATE analysis. Through the GSVA package in R software, ssGSEA was used to quantify the infiltration levels of immune cell types (http://www.bioconductor.org/packages/release/bioc/html/GSVA.html). The limma R package was used to identify DEGs. DEGs were identified by comparing gene expressions between low and high score groups using the criteria: mRNA expression values of |log2 FC| > 1 and FDR < 0.05. GO and KEGG enrichment analyses were performed using clusterProfiler and DOSE packages. Hierarchical clustering of LUAD was performed using the sparcl package. Survival analyses were performed using Kaplan-Meier survival analysis while log-rank tests were performed using survival R package. Comparisons of prognostic gene expressions between groups were performed by the *T* test. *P* ≤ 0.05 was the threshold for statistical significance.

## 3. Results

### 3.1. Immune Scores Were Significantly Associated with LUAD Clinical Stages

**Figure 1:**
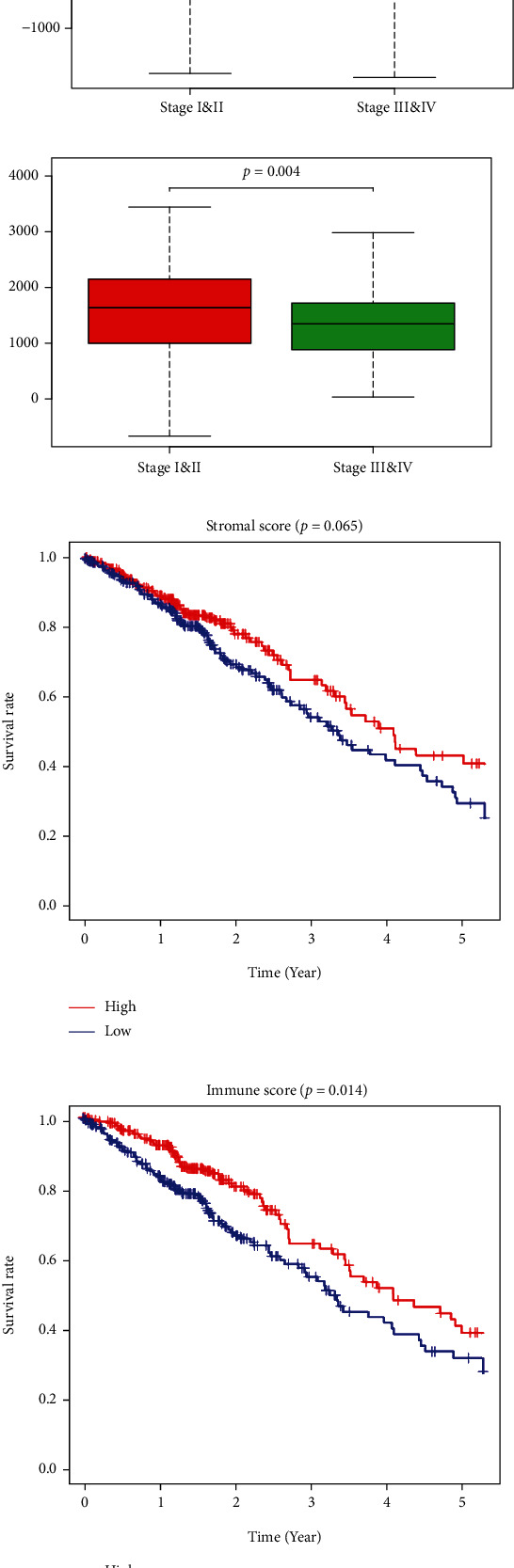
ESTIMATE scores in clinical stages and survival curves. (a) Comparison of stromal scores across clinical stages. (b) Comparison of immune scores across clinical stages. (c) Survival curve of stromal scores. (d) Survival curve of immune scores. ESTIMATE: Estimation of STromal and Immune cells in MAlignant Tumor tissues using Expression data.

Data on a cohort of 522 LUAD cases with mRNA expression profiles and corresponding clinical data were downloaded from TCGA. Of these, 280 cases were female while 242 male. In this analysis, immune scores ranged from -942.51 to 3442.08 while stromal scores ranged from -1789.62 to 2097.96. In various LUAD stages, immune scores were significantly high in stages I and II, relative to stages III and IV, indicating higher TME immune infiltrations in stages I and II (Figure 1(b)). There were no significant differences in stromal scores among different stages (Figure 1(a)). Clinical characteristics for all cases in this study are shown in Table S2.

### 3.2. Immune Score as a Potential Prognostic Marker for LUAD

To determine the effects of ESTIMATE scores on overall survival, cases were assigned into high and low score groups based on median ESTIMATE scores. Differences in survival time between high and low ESTIMATE score groups are presented using survival curves. Immune score analysis revealed a high survival rate, relative to the high score within five years (*P* = 0.019 in log-rank test, Figure 1(d)). A similar trend was observed for stromal scores, although the differences were insignificant (*P* = 0.099) (Figure 1(c)).

### 3.3. Differentially Expressed Genes with Immune and Stromal Score Groups in LUAD

**Figure 2:**
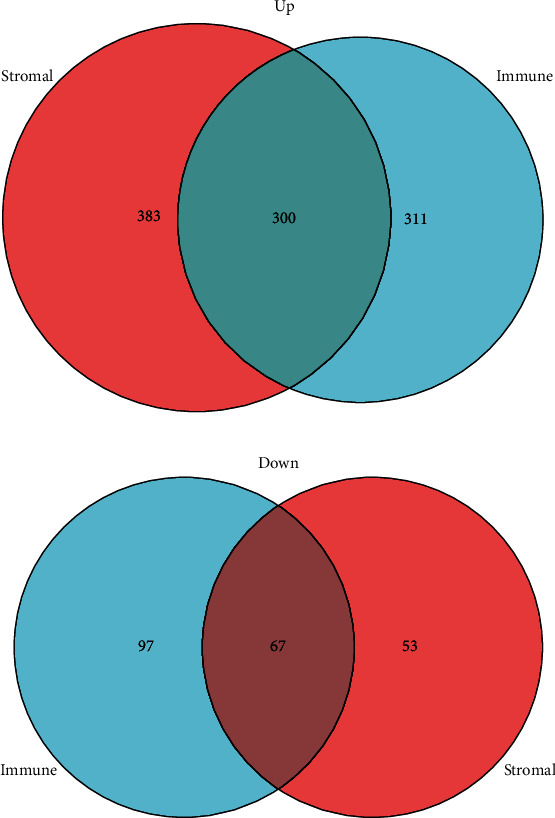
Venn diagram of DEGs based on immune and stromal scores. (a) Venn diagram showing upregulated DEGs. (b) Venn diagram showing downregulated DEGs. DEGs: differentially expressed genes.

Venn diagram analysis was used to identify co-DEGs in immune and stromal categories (Figure 2). There were 300 simultaneous upregulated genes in high score groups based on immune and stromal scores and 67 downregulated genes.

### 3.4. GO and KEGG Enrichment Analysis

**Figure 3:**
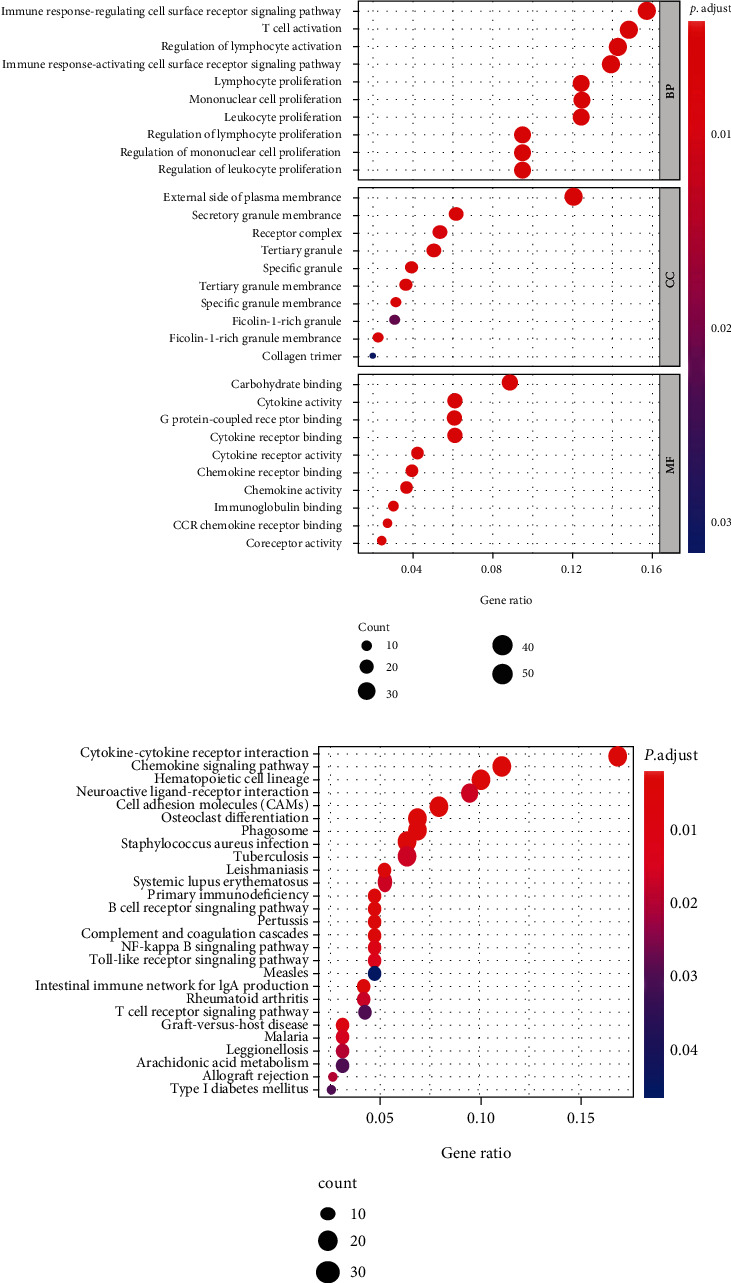
GO and KEGG pathway analysis for DEGs based on TME scoring model. (a) GO term enrichment; top ten pathways of each category with *P* < 0.05 are shown. (b) KEGG pathway enrichment. Top thirty pathways with *P* < 0.05 are shown. GO: Gene Ontology; KEGG: Kyoto Encyclopedia of Genes and Genomes.

To explore the functions of DEGs in LUAD, GO term and KEGG analyses were performed using R packages. Significantly enriched GO terms for DEGs were in BPs of immune responses regulating signaling pathways, lymphocyte proliferation, and mononuclear cell proliferation. Enriched MF processes included carbohydrate binding, immunoglobulin binding, and chemokine activity. Enriched CCs included external side of the plasma membrane, tertiary granule membrane, and tertiary granule (Figure 3(a)). Go term analysis revealed that the DEGs were predominantly correlated with immune functions. These findings are in agreement with previous reports that immune cells and the extracellular matrix are involved in lung TME [15]. KEGG pathway analysis indicated that DEGs were mainly enriched in cytokine-cytokine receptor interactions, hematopoietic cell lineage, and chemokine signaling pathway (Figure 3(b)).

### 3.5. Survival Analysis with Gene Expressions of DEGs

**Figure 4:**
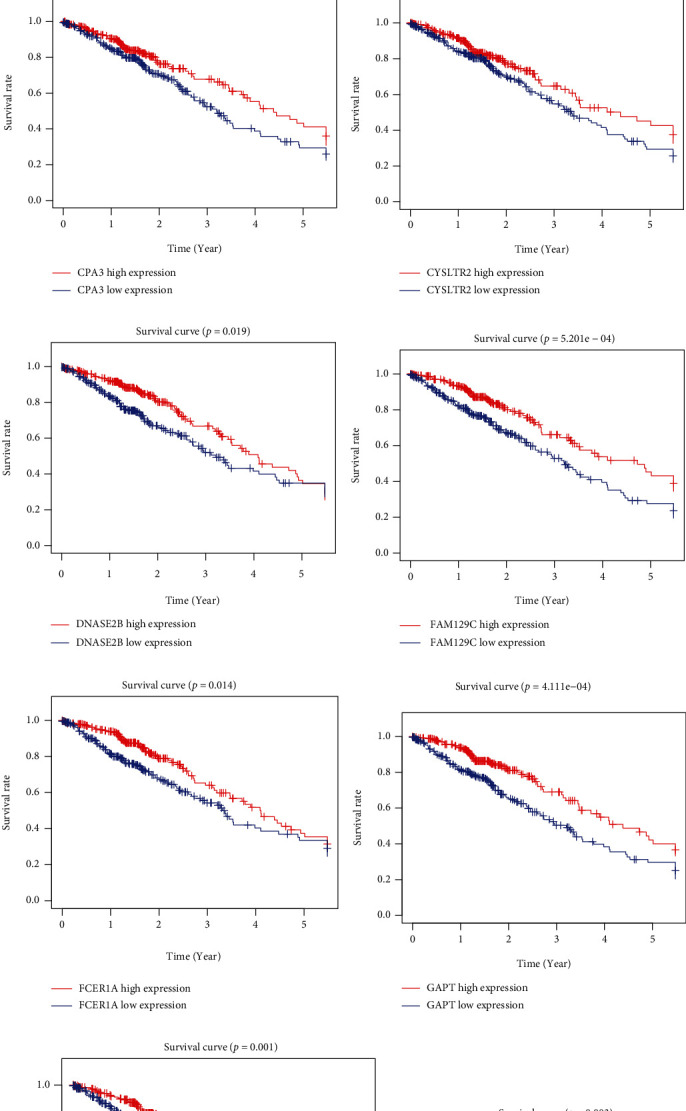
Prognostic value of mRNA expression (Kaplan-Meier plotter) of DEGs based on TME scoring model in LUAD patients. (a–n) Show the relation of mRNA expression of DEGs with the prognosis in LUAD patients from TCGA cohort using Kaplan-Meier plotter. Kaplan-Meier survival curves were generated for selected DEGs from high (red line) and low (blue line) gene expression groups. DEGs: differentially expressed genes; TME: tumor microenvironment.

**Figure 5:**
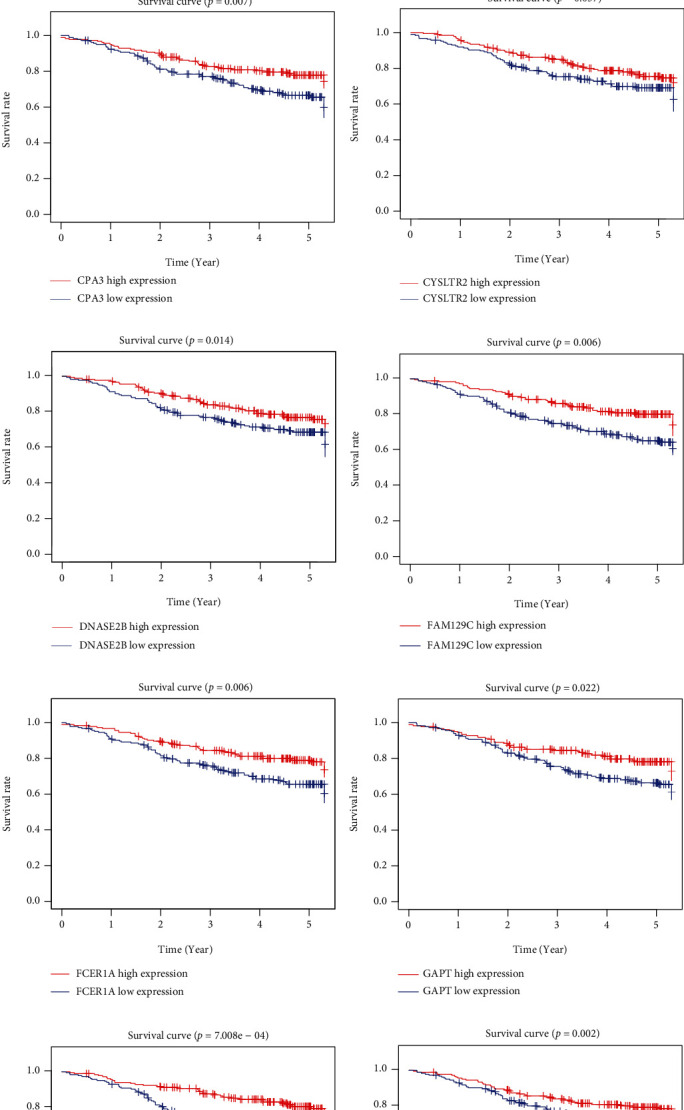
Prognostic value of mRNA expression (Kaplan-Meier plotter) of DEGs based on TME scoring model in LUAD patients. (a–n) Show the relation of mRNA expression of DEGs with the prognosis in LUAD patients from GEO cohort using Kaplan-Meier plotter. Kaplan-Meier survival curves were generated for selected DEGs from high (red line) and low (blue line) gene expression groups. DEGs: differentially expressed genes; TME: tumor microenvironment; DEGs: differentially expressed genes.

To elucidate on the effects of DEGs on overall survival outcomes of LUAD patients, TCGA database was used to generate Kaplan-Meier survival curves. *P* ≤ 0.05 indicated significant differences in survival outcomes. Among the 367 DEGs, 119 were significantly correlated with survival time, as revealed by log-rank test (Table S3). To validate the observations made in TCGA LUAD cohort in a different cohort, we analyzed the gene expression data for 369 LUAD cases in GEO. It was validated that 66 genes were significantly associated with LUAD prognosis (Table S4), while 14 genes were at the intersection of TCGA and GEO databases, which have relationships with survival (Figures 4 and 5).

### 3.6. Immune Landscape of Immune Clusters

**Figure 6:**
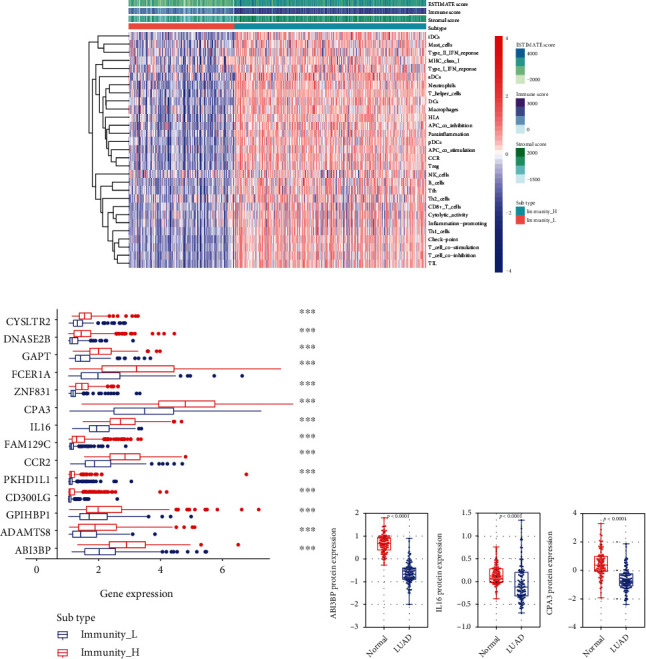
Immune landscape of immune subtypes. (a) Stromal score and immune score in different immune subtypes. The red and blue colours used on the heat map indicate the high and low relative activity of immune cells, respectively. (b) Comparison of prognostic gene expression levels between immune subtypes. ^∗^*P* < 0.05, ^∗∗^*P* < 0.01, and ^∗∗∗^*P* < 0.001. (c) Comparisons of the expression at protein level of the three genes between lung adenocarcinoma (LUAD) and normal tissues in CPTAC. CPTAC: the Clinical Proteomic Tumor Analysis Consortium.

Based on ESTIMATE and ssGSEA, immune characteristics of immune subtypes were visualized in the heat map (Figure 6(a)). Immune and stromal scores were significantly high in Immunity_H subtype and low in Immunity_L subtype. This indicated that the finding from ESTIMATE analysis was consistent with ssGSEA. Furthermore, expressions of the 14 prognosis-associated genes were compared in different immune subtypes. Expressions of most of these genes significantly increased from Immunity_L subtype to Immunity_H subtype (all *P* < 0.001; Figure 6(b)).

### 3.7. Differential Expressions of Prognostic Genes at the Protein Level

**Figure 7:**
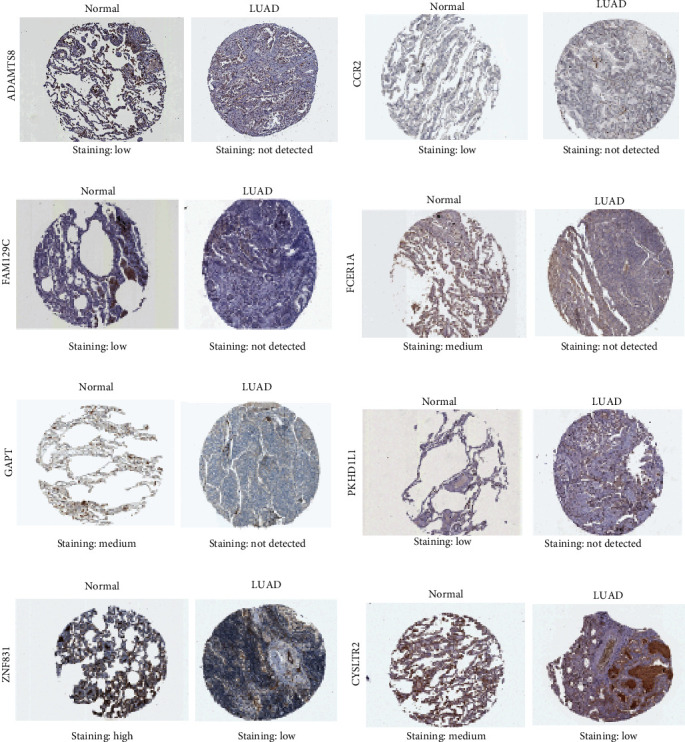
Relative immunohistochemistry results of 8 prognostic genes in LUAD tissues and normal lung tissues form the Human Protein Atlas database. The protein expression levels of 8 prognosis-related DEGs in LUAD and normal lung tissues by IHC images.

In the CPTAC database, ABI3BP, IL16, and CPA3 were found to be significantly downregulated in LUAD samples (Figure 6(c)). In the HPA database, protein expressions of the seven genes (ADAMTS8, CCR2, CYSLTR2, FAM129C, FCER1A, GAPT, PKHD1L1, and ZNF831) were markedly low in tumor tissues with less intense antibody staining and fewer stained cells in LUAD (Figure 7). GPIHBP1, CD300LG, and DNASE2B were not shown in either CPTAC or HPA databases.

## 4. Discussion

In this study, we identified 14 tumor microenvironment-related prognostic genes in lung adenocarcinoma from TCGA and GEO databases. Based on enrichment levels of immune cell types, we clustered LUAD into Immunity_H and Immunity_L subtypes. These genes were upregulated in Immunity_H subtype, indicating that they were closely associated with immune cell infiltrations in LUAD.

LUAD is often diagnosed in advanced stages. It is characterized by high metastasis and poor prognosis. Despite advances in LUAD treatment, long-term prognosis remains poor. In recent years, efforts have been made to identify potential prognostic markers for LUAD. For instance, the prognostic potential of LUAD gene expression signatures has been extensively studied [16].

Currently, TNM staging is the main basis for determining lung cancer prognosis. However, its effectiveness is limited by the fact that clinical outcomes for different patients at the same TNM stage can vary significantly [17]. Disease progression is influenced by immune cell infiltrations in the TME. In several cancers, immune-related indicators correlate better with clinical outcomes, relative to TNM staging [18–20]. Therefore, immune scores associated with TME are essential components of the staging system [21]. Development of immune checkpoint inhibitors, including antibodies against the PD-1/PD-L1, has greatly improved cancer treatment and outcomes [22], especially for NSCLC [23]. Various studies have revealed the TME of NSCLC consists of various immune cells, indicating the prognostic value of TME immune cells [24]. In this study, we determined whether ESTIMATE scores of TME are indicative of overall survival and investigated the prognostic value of stromal and immune infiltration scores in LUAD. These scores predict patient clinical outcomes. Higher immune scores were associated with better LUAD prognosis, consistent with previous findings [25–27]. Tumors with high immune cell infiltrations in the TME exhibit favorable prognosis [28]. Our data indicates that immune scores reflect survival and clinical outcomes in LUAD. It should be determined whether ESTIMATE immune scores are potential prognostic biomarkers.

The prognostic potential of stromal and immune scores has been determined for multiple cancers [29, 30]. Immune and stromal cells are the most important nontumor components in tumor tissues. Lung tissues contain infiltrating immune cells from innate and acquired immune components. The type, concentration, and localization of these immune factors may indicate prognostic outcomes for cancers and other pathologies. Tumor cells and other TME constituents secrete chemokines and chemokine receptors [31], which modulate the proliferation and invasion of malignant cells. The association between immune scores obtained by ESTIMATE analysis and disease prognosis across cancer types has been determined. Higher scores indicate better prognosis for breast cancer, melanoma, and ovarian cancer but poor prognosis for hepatocellular carcinoma [30]. The lung cancer TME is rich in immune cells [32]. In LUAD, high immune scores and high infiltrations by adaptive immune cells are associated with favorable outcomes.

FPR2 is a G protein coupled receptor (GPCR) that plays an important role in antibacterial inflammation. Upregulated FPR2 suppresses epithelial-mesenchymal transition of lung cancer cells [33]. Interestingly, certain genotypes of interleukins can predict the risk of death and progression, in NSCLC patients [34]. However, the mechanisms through which interleukins influence prognosis in NSCLC have not been established. We established that CCR2 is a hub and prognostic gene. CCR2 recruits precursors for exudative macrophages and inflammatory DCs into the lung. Until recently, biological functions of CCR2 in lung cancer had yet to be established. CCR2 induces macrophage and cancer cell crosstalk, an essential mechanism for driving lung cancer progression [35]. CCR2 is involved in promotion of tumor-supportive immune microenvironment [36]. In this study, elevated CCR2 levels correlated with longer survival time, consistent with previous findings [37]. Therefore, the role of CCR2 in lung cancer requires further investigations.

Although our study explored the prognostic genes based on TME of LUAD, their prognostic significance as well as the involved mechanisms were not determined. Moreover, our findings are based on bioinformatics analyses, and further validation should be performed in future studies.

This study elucidates on the prognostic potential of TME in LUAD and provides the foundation for further studies on prognostic biomarkers in LUAD.

## Data Availability

The data used to support the findings of this study are available from the corresponding authors upon request. All data used in this study can be downloaded from TCGA (https://portal.gdc.cancer.gov/), GEO (https://www.ncbi.nlm.nih.gov/geo/), CPTAC (https://proteomics.cancer.gov/programs/cptac), and HPA (https://www.proteinatlas.org) databases.

## Supplementary Materials

Supplementary 1 Figure S1: whole procedure for analyzing prognostic genes and immune landscape signatures based on tumor microenvironment in lung adenocarcinoma.

Supplementary 2 Table S1: marker genes for 29 immune cell subtypes.

Supplementary 3 Table S2: clinical characteristics of LUAD patients from TCGA and GEO cohorts.

Supplementary 4 Table S3: DEGs with significant correlation with overall survival of LUAD.

Supplementary 5 Table S4: DEGs with significant correlation with overall survival of LUAD data from GEO.
