# Supplementary figures and images for: Identification of Prognostic Genes and Immune Landscape Signatures Based on Tumor Microenvironment in Lung Adenocarcinoma
Source: Dis Markers. 2022 Aug 18;2022:6703053. doi: 10.1155/2022/6703053 (PMC9411923; doi:10.1155/2022/6703053)

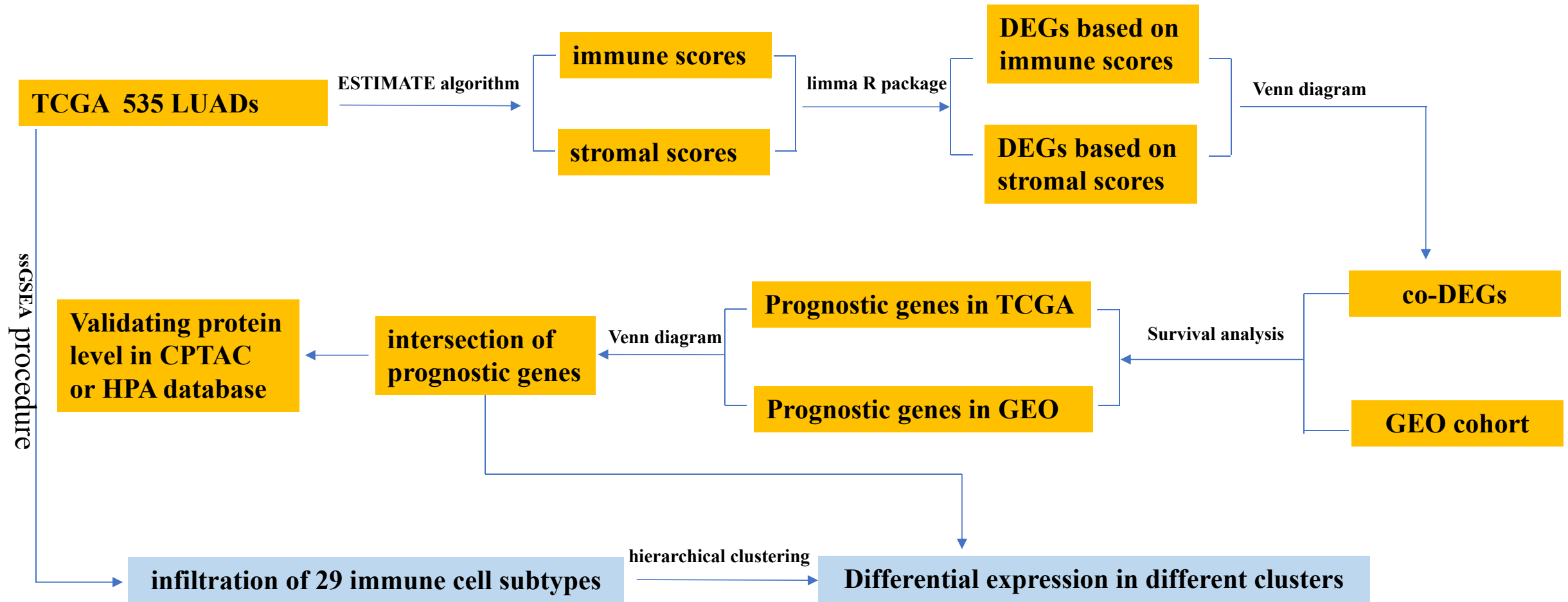

Supplement: Supplementary 1 — Figure S1: whole procedure for analyzing prognostic genes and immune landscape signatures based on tumor microenvironment in lung adenocarcinoma. [file 6703053.f1.pdf]
