# Supplementary material for: Identification of Prognostic Genes and Immune Landscape Signatures Based on Tumor Microenvironment in Lung Adenocarcinoma
Source: Dis Markers. 2022 Aug 18;2022:6703053. doi: 10.1155/2022/6703053 (PMC9411923; doi:10.1155/2022/6703053)
Supplement: Supplementary 2 — Table S1: marker genes for 29 immune cell subtypes. [file 6703053.f2.docx]

| **Supplementary Table S1. Marker genes for 29 immune cell** **subtypes** | |
| --- | --- |
| immune cell subtypes | marker genes |
| aDCs | CD83,LAMP3,CCL1 |
| APC_co_inhibition | C10orf54,CD274,LGALS9,PDCD1LG2,PVRL3 |
| APC_co_stimulation | CD40,CD58,CD70,ICOSLG,SLAMF1,TNFSF14,TNFSF15,TNFSF18,TNFSF4,TNFSF8,TNFSF9 |
| B_cells | BACH2,BANK1,BLK,BTLA,CD79A,CD79B,FCRL1,FCRL3,HVCN1,RALGPS2 |
| CCR | CCL16,TPO,TGFBR2,CXCL2,CCL14,TGFBR3,IL11RA,CCL11,IL4I1,IL33,CXCL12,CXCL10,BMPER,BMP8A,CXCL11,IL21R,IL17B,TNFRSF9,ILF2,CX3CR1,CCR8,TNFSF12,CSF3,TNFSF4,BMP3,CX3CL1,BMP5,CXCR2,TNFRSF10D,BMP2,CXCL14,CCL28,CXCL3,BMP6,CCL21,CXCL9,CCL23,IL6,TNFRSF18,IL17RD,IL17D,IL27,CCL7,IL1R1,CXCR4,CXCR2P1,TGFB1I1,IFNGR1,IL9R,IL1RAPL1,IL11,CSF1,IL20RA,IL25,TNFRSF4,IL18,ILF3,CCL20,TNFRSF12A,IL6ST,CXCL13,IL12B,TNFRSF8,IL6R,BMPR2,IFNE,IL1RAPL2,IL3RA,BMP4,CCL24,TNFSF13B,CCR4,IL2RA,IL32,TNFRSF10C,IL22RA1,BMPR1A,CXCR5,CXCR3,IFNA8,IL17REL,IFNB1,IFNAR1,TNFRSF1B,CCL17,IFNL1,IL16,IL1RL1,ILK,CCL25,ILDR2,CXCR1,IL36RN,IL34,TGFB1,IFNG,IL19,ILKAP,BMP2K,CCR10,ILDR1,EPO,CCR7,IL17C,IL23A,CCR5,IL7,EPOR,CCL13,IL2RG,IL31RA,TNFAIP6,IFNL2,BMP1,IL12RB1,TNFAIP8,IL4R,TNFRSF6B,TNFAIP8L1,TNFRSF10B,IFNL3,CCL5,CXCL6,CXCL1,CCR3,TNFSF11,CSF1R,IL21,IL1RAP,IL12RB2,CCL1,IL17RA,CCR1,IL1RN,TNFRSF11B,TNFRSF14,IL13,IL2RB,BMP8B,CCL2,IL24,IL18RAP,TGFBI,TNFSF10,TNFRSF11A,CXCL5,IL5RA,TNFSF9,IL1RL2,TNFRSF13C,IL36G,IL15RA,TNFRSF21,CXCL8,IL22RA2,TNFAIP8L2,IL18R1,IFNLR1,CXCR6,CCL3L3,TNFRSF1A,IL17RE,IFNGR2,IL17RC,TNFAIP8L3,ILVBL,TGFBRAP1,CCL4L1,CSF2RA,CCRN4L,CCL26,TNFAIP1,CCRL2,IFNA10,TNFRSF17,IFNA13,IL20,IL18BP,CCL3L1,TNFSF12-TNFSF13,IL5,IL23R,IL26,TNF,TGFA,CSF2,IL1F10,CXCL17,TNFSF13,IFNA4,IL37,IL12A,IL7R,IFNA1,IL1A,IL4,IL2,CCL22,CSF3R,IL10,IFNK,TGFB2,IL1R2,IL1B,IL17F,IL27RA,IL15,TNFSF8,IL36B,XCL1,CXCL16,TNFRSF19,IL3,CCL3,IFNA2,BMPR1B,IFNA21,TNFSF18,CCL8,IL17RB,TNFRSF25,IL22,IL10RB,IFNAR2,CCL18,IFNA16,CSF2RB,IL36A,TNFAIP3,IL13RA2,IL13RA1,CCR9,TNFRSF10A,IFNA7,IFNW1,XCL2,TNFSF14,CCR2,BMP15,BMP10,CCL15-CCL14,TGFBR1,IFNA5,BMP7,IFNA14,IL20RB,IL10RA,IFNA17,CCR6,TGFB3,CCL15,CCL4,CCL27,TNFRSF13B,TNFAIP2,IL31,IL17A,TNFSF15,CCL19,IFNA6,IL9 |
| CD8+_T_cells | CD8A |
| Check-point | IDO1,LAG3,CTLA4,TNFRSF9,ICOS,CD80,PDCD1LG2,TIGIT,CD70,TNFSF9,ICOSLG,KIR3DL1,CD86,PDCD1,LAIR1,TNFRSF8,TNFSF15,TNFRSF14,IDO2,CD276,CD40,TNFRSF4,TNFSF14,HHLA2,CD244,CD274,HAVCR2,CD27,BTLA,LGALS9,TMIGD2,CD28,CD48,TNFRSF25,CD40LG,ADORA2A,VTCN1,CD160,CD44,TNFSF18,TNFRSF18,BTNL2,C10orf54,CD200R1,TNFSF4,CD200,NRP1 |
| Cytolytic_activity | PRF1,GZMA |
| DCs | CCL17,CCL22,CD209,CCL13 |
| HLA | ,HLA-E,HLA-DPB2,HLA-C,HLA-J,HLA-DQB1,HLA-DQB2,HLA-DQA2,HLA-DQA1,HLA-A,HLA-DMA,HLA-DOB,HLA-DRB1,HLA-H,HLA-B,HLA-DRB5,HLA-DOA,HLA-DPB1,HLA-DRA,HLA-DRB6,HLA-L,HLA-F,HLA-G,HLA-DMB,HLA-DPA1 |
| iDCs | CD1A,CD1E |
| Inflammation-promoting | CCL5,CD19,CD8B,CXCL10,CXCL13,CXCL9,GNLY,GZMB,IFNG,IL12A,IL12B,IRF1,PRF1,STAT1,TBX21 |
| Macrophages | C11orf45,CD68,CLEC5A,CYBB,FUCA1,GPNMB,HS3ST2,LGMN,MMP9,TM4SF19 |
| Mast_cells | CMA1,MS4A2,TPSAB1 |
| MHC_class_I | B2M,HLA-A,TAP1 |
| Neutrophils | EVI2B,HSD17B11,KDM6B,MEGF9,MNDA,NLRP12,PADI4,SELL,TRANK1,VNN3 |
| NK_cells | KLRC1,KLRF1 |
| Parainflammation | CXCL10,PLAT,CCND1,LGMN,PLAUR,AIM2,MMP7,ICAM1,MX2,CXCL9,ANXA1,TLR2,PLA2G2D,ITGA2,MX1,HMOX1,CD276,TIRAP,IL33,PTGES,TNFRSF12A,SCARB1,CD14,BLNK,IFIT3,RETNLB,IFIT2,ISG15,OAS2,REL,OAS3,CD44,PPARG,BST2,OAS1,NOX1,PLA2G2A,IFIT1,IFITM3,IL1RN |
| pDCs | CLEC4C,CXCR3,GZMB,IL3RA,IRF7,IRF8,LILRA4,PHEX,PLD4,PTCRA |
| T_cell_co-inhibition | BTLA,C10orf54,CD160,CD244,CD274,CTLA4,HAVCR2,LAG3,LAIR1,TIGIT |
| T_cell_co-stimulation | CD2,CD226,CD27,CD28,CD40LG,ICOS,SLAMF1,TNFRSF18,TNFRSF25,TNFRSF4,TNFRSF8,TNFRSF9,TNFSF14 |
| T_helper_cells | CD4 |
| Tfh | PDCD1,CXCL13,CXCR5 |
| Th1_cells | IFNG,TBX21,CTLA4,STAT4,CD38,IL12RB2,LTA,CSF2 |
| Th2_cells | PMCH,LAIR2,SMAD2,CXCR6,GATA3,IL26 |
| TIL | ITM2C,CD38,THEMIS2,GLYR1,ICOS,F5,TIGIT,KLRD1,IRF4,PRKCQ,FCRL5,SIRPG,LPXN,IL2RG,CCL5,LCK,TRAF3IP3,CD86,MAL,LILRB1,DOK2,CD6,PAG1,LAX1,PLEK,PIK3CD,SLAMF1,XCL1,GPR171,XCL2,TBX21,CD2,CD53,KLHL6,SLAMF6,CD40,SIT1,TNFRSF4,CD79A,CD247,LCP2,CD3D,CD27,SH2D1A,FYB,ARHGAP30,ACAP1,CST7,CD3G,IL2RB,CD3E,FCRL3,CORO1A,ITK,TCL1A,CYBB,CSF2RB,IKZF1,NCF4,DOCK2,CCR2,PTPRC,PLAC8,NCKAP1L,IL7R,6-Sep,CD28,STAT4,CD8A,LY9,CD48,HCST,PTPRCAP,SASH3,ARHGAP25,LAT,TRAT1,IL10RA,PAX5,CCR7,DOCK11,PARVG,SPNS1,CD52,HCLS1,ARHGAP9,GIMAP6,PRKCB,MS4A1,GPR18,TBC1D10C,GVINP1,P2RY8,EVI2B,VAMP5,KLRK1,SELL,MPEG1,MS4A6A,ARHGAP15,MFNG,GZMK,SELPLG,TARP,GIMAP7,FAM65B,INPP5D,ITGA4,MZB1,GPSM3,STK10,CLEC2D,IL16,NLRC3,GIMAP5,GIMAP4,IFFO1,CFH,PVRIG,CFHR1 |
| Treg | IL12RB2,TMPRSS6,CTSC,LAPTM4B,TFRC,RNF145,NETO2,ADAT2,CHST2,CTLA4,NFE2L3,LIMA1,IL1R2,ICOS,HSDL2,HTATIP2,FKBP1A,TIGIT,CCR8,LTA,SLC35F2,IL21R,AHCYL1,SOCS2,ETV7,BCL2L1,RRAGB,ACSL4,CHRNA6,BATF,LAX1,ADPRH,TNFRSF4,ANKRD10,CD274,CASP1,LY75,NPTN,SSTR3,GRSF1,CSF2RB,TMEM184C,NDFIP2,ZBTB38,ERI1,TRAF3,NAB1,HS3ST3B1,LAYN,JAK1,VDR,LEPROT,GCNT1,PTPRJ,IKZF2,CSF1,ENTPD1,TNFRSF18,METTL7A,KSR1,SSH1,CADM1,IL1R1,ACP5,CHST7,THADA,CD177,NFAT5,ZNF282,MAGEH1 |
| Type_I_IFN_Reponse | DDX4,IFIT1,IFIT2,IFIT3,IRF7,ISG20,MX1,MX2,RSAD2,TNFSF10 |
| Type_II_IFN_Reponse | GPR146,SELP,AHR |
