# Supplementary material for: Identification of Prognostic Genes and Immune Landscape Signatures Based on Tumor Microenvironment in Lung Adenocarcinoma
Source: Dis Markers. 2022 Aug 18;2022:6703053. doi: 10.1155/2022/6703053 (PMC9411923; doi:10.1155/2022/6703053)
Supplement: Supplementary 3 — Table S2: clinical characteristics of LUAD patients from TCGA and GEO cohorts. [file 6703053.f3.docx]

| **Table S2 Clinical Characteristics of LUAD patients from TCGA and GEO cohorts.** | | | | |
| --- | --- | --- | --- | --- |
|  | TCGA | GSE3141 | GSE31210 | GSE30219 |
| Age, n (%) |  |  |  |  |
| <65 years | 223 (42.7%) | - | 164 (72.6%) | 55 (64.7%) |
| ≥65 years | 280 (53.6%) | - | 62 (27.4%) | 30 (35.3%) |
| Unknown | 19 (3.6%) | - |  |  |
| Gender, n (%) |  |  |  |  |
| Female | 280 (53.6%) | - | 121 (53.5%) | 19 (22.4%) |
| Male | 242 (46.4%) | - | 105 (46.5%) | 66 (77.6%) |
| Stage, n (%) |  |  |  |  |
| I | 279 (53.4%) | - | 168 (74.3%) | - |
| II | 124 (23.8%) | - | 58 (25.7%) | - |
| III | 85 (16.3%) | - | 0 | - |
| IV | 26 (5.0%) | - | 0 | - |
| UnKnown | 8 (1.5%) |  |  |  |
| Event, n(%) |  |  |  |  |
| Dead | 187 (35.8) | 32 (55.2%) | 35 (15.5%) | 45 (52.9%) |
| Alive | 335 (64.2) | 26 (44.8%) | 191 (84.5%) | 40 (47.1%) |
| There were 13 patients without any clinical information in TCGA cohort. | | | | |
