# Supplementary material for: Identification of Prognostic Genes and Immune Landscape Signatures Based on Tumor Microenvironment in Lung Adenocarcinoma
Source: Dis Markers. 2022 Aug 18;2022:6703053. doi: 10.1155/2022/6703053 (PMC9411923; doi:10.1155/2022/6703053)
Supplement: Supplementary 4 — Table S3: DEGs with significant correlation with overall survival of LUAD. [file 6703053.f4.docx]

**Supplementary Table S3. DEGs whose expression is significant in overall survival of LUAD.**

|  | gene | pvalue |
| --- | --- | --- |
| 1 | GAPT | 0.000402 |
| 2 | FAM129C | 0.000626 |
| 3 | TMEM273 | 0.000698 |
| 4 | FCRL1 | 0.000941 |
| 5 | CLEC17A | 0.000966 |
| 6 | CLEC10A | 0.000981 |
| 7 | ABCC8 | 0.00114 |
| 8 | RUBCNL | 0.001188 |
| 9 | CD200R1 | 0.001253 |
| 10 | P2RY12 | 0.001415 |
| 11 | PRKCB | 0.001494 |
| 12 | GPIHBP1 | 0.001716 |
| 13 | TLR7 | 0.001761 |
| 14 | LILRA4 | 0.001763 |
| 15 | CD33 | 0.001981 |
| 16 | SCIMP | 0.002099 |
| 17 | TESPA1 | 0.002158 |
| 18 | RCSD1 | 0.002306 |
| 19 | TLR10 | 0.00242 |
| 20 | PKHD1L1 | 0.002502 |
| 21 | STAP1 | 0.002549 |
| 22 | BTK | 0.002682 |
| 23 | CCR2 | 0.002949 |
| 24 | FDCSP | 0.00339 |
| 25 | MS4A1 | 0.00345 |
| 26 | GIMAP8 | 0.003453 |
| 27 | ARHGAP15 | 0.00371 |
| 28 | INHA | 0.005011 |
| 29 | SLAMF1 | 0.005013 |
| 30 | IL16 | 0.00516 |
| 31 | CD80 | 0.005905 |
| 32 | CHRNA5 | 0.005937 |
| 33 | RTN1 | 0.005967 |
| 34 | FCRL3 | 0.005992 |
| 35 | TNFRSF13B | 0.006339 |
| 36 | IRF8 | 0.006727 |
| 37 | PTPRQ | 0.006843 |
| 38 | ARHGEF6 | 0.006945 |
| 39 | FCGR1B | 0.006964 |
| 40 | CLEC4G | 0.007368 |
| 41 | CD19 | 0.007948 |
| 42 | SIT1 | 0.008539 |
| 43 | CR1 | 0.009009 |
| 44 | RASGRP4 | 0.009017 |
| 45 | FOLR2 | 0.009257 |
| 46 | SPN | 0.009354 |
| 47 | ABI3BP | 0.00936 |
| 48 | S100P | 0.010032 |
| 49 | GIMAP4 | 0.010101 |
| 50 | DOK2 | 0.010252 |
| 51 | CCL14 | 0.011312 |
| 52 | CRB2 | 0.01159 |
| 53 | LST1 | 0.012262 |
| 54 | BLK | 0.012558 |
| 55 | CD300C | 0.012675 |
| 56 | APOC4-APOC2 | 0.01275 |
| 57 | XIRP1 | 0.013161 |
| 58 | CLEC4A | 0.013235 |
| 59 | ALOX5AP | 0.013304 |
| 60 | FCRLA | 0.013307 |
| 61 | CD1E | 0.014098 |
| 62 | HLA-DQA1 | 0.014135 |
| 63 | PTX3 | 0.014138 |
| 64 | PI16 | 0.014197 |
| 65 | CD79B | 0.01434 |
| 66 | TNFSF8 | 0.014459 |
| 67 | PLEK | 0.014664 |
| 68 | MPEG1 | 0.014711 |
| 69 | NCKAP1L | 0.015135 |
| 70 | TMEM236 | 0.015264 |
| 71 | FCER1A | 0.015518 |
| 72 | GNRH2 | 0.016385 |
| 73 | NLRC4 | 0.016582 |
| 74 | PTPRC | 0.016672 |
| 75 | DNASE2B | 0.017054 |
| 76 | ICAM3 | 0.017508 |
| 77 | CD53 | 0.017735 |
| 78 | P2RY13 | 0.018308 |
| 79 | RSPO1 | 0.019362 |
| 80 | HPGDS | 0.019483 |
| 81 | LY86 | 0.019529 |
| 82 | GIMAP6 | 0.020121 |
| 83 | MS4A6A | 0.020353 |
| 84 | ADAMTS8 | 0.020635 |
| 85 | IKZF1 | 0.020857 |
| 86 | CYTH4 | 0.0212 |
| 87 | SASH3 | 0.021518 |
| 88 | ACKR1 | 0.022933 |
| 89 | CLECL1 | 0.023264 |
| 90 | FCRL4 | 0.023437 |
| 91 | EVI2B | 0.02493 |
| 92 | BCAN | 0.025019 |
| 93 | KBTBD8 | 0.025335 |
| 94 | CD226 | 0.025549 |
| 95 | SLCO2B1 | 0.027407 |
| 96 | CSF2RB | 0.027971 |
| 97 | OLR1 | 0.028903 |
| 98 | CD22 | 0.030414 |
| 99 | LAX1 | 0.031508 |
| 100 | CD300LG | 0.031578 |
| 101 | MCEMP1 | 0.03297 |
| 102 | FCER2 | 0.033155 |
| 103 | CNR2 | 0.033924 |
| 104 | DOCK2 | 0.03433 |
| 105 | BARX1 | 0.034413 |
| 106 | GPR174 | 0.036079 |
| 107 | MARCH1 | 0.037152 |
| 108 | GPR34 | 0.038493 |
| 109 | CD28 | 0.038735 |
| 110 | GIMAP5 | 0.039342 |
| 111 | SERTM1 | 0.041586 |
| 112 | CASS4 | 0.04257 |
| 113 | RGS18 | 0.043087 |
| 114 | LCN6 | 0.043664 |
| 115 | ZNF831 | 0.04378 |
| 116 | CPA3 | 0.04439 |
| 117 | CD69 | 0.045479 |
| 118 | ADPRHL1 | 0.047125 |
| 119 | VEGFD | 0.049025 |
