# Supplementary material for: Identification of Prognostic Genes and Immune Landscape Signatures Based on Tumor Microenvironment in Lung Adenocarcinoma
Source: Dis Markers. 2022 Aug 18;2022:6703053. doi: 10.1155/2022/6703053 (PMC9411923; doi:10.1155/2022/6703053)
Supplement: Supplementary 5 — Table S4: DEGs with significant correlation with overall survival of LUAD data from GEO. [file 6703053.f5.docx]

**Supplementary Table S4. DEGs whose expression is significant in overall survival of LUAD from GEO.**

|  | gene | pvalue |
| --- | --- | --- |
| 1 | ABCD2 | 0.014968 |
| 2 | ABI3BP | 7.36E-05 |
| 3 | ADAMDEC1 | 0.011508 |
| 4 | ADAMTS8 | 0.00062 |
| 5 | ADH1B | 1.73E-06 |
| 6 | AQP9 | 0.011621 |
| 7 | BCL2A1 | 0.007917 |
| 8 | BPIFA2 | 0.029229 |
| 9 | C1QB | 0.001624 |
| 10 | C1QC | 0.010074 |
| 11 | C3AR1 | 0.001242 |
| 12 | C7 | 0.006973 |
| 13 | CASP5 | 0.043489 |
| 14 | CCR2 | 0.005072 |
| 15 | CD180 | 0.000544 |
| 16 | CD300LG | 0.002512 |
| 17 | CD86 | 0.006214 |
| 18 | CLEC4C | 0.005754 |
| 19 | COL6A5 | 0.003043 |
| 20 | COL6A6 | 0.00424 |
| 21 | CPA3 | 0.011064 |
| 22 | CRB2 | 0.021158 |
| 23 | CSF1R | 0.005099 |
| 24 | CYSLTR2 | 0.041061 |
| 25 | DACT2 | 0.000119 |
| 26 | DKK4 | 0.006562 |
| 27 | DNASE2B | 0.043752 |
| 28 | DOCK2 | 0.00741 |
| 29 | ENO3 | 0.010525 |
| 30 | FAM129C | 0.006399 |
| 31 | FCER1A | 0.028643 |
| 32 | FCGR1B | 0.02132 |
| 33 | FCGR2B | 0.042824 |
| 34 | FMO2 | 0.02372 |
| 35 | FPR1 | 0.010768 |
| 36 | FPR3 | 0.008588 |
| 37 | GAPT | 0.038223 |
| 38 | GPIHBP1 | 0.00121 |
| 39 | GPR84 | 0.000981 |
| 40 | HAVCR2 | 0.015126 |
| 41 | HMGCS2 | 0.003083 |
| 42 | IL16 | 0.008734 |
| 43 | IL1B | 0.031244 |
| 44 | IL21R | 0.042042 |
| 45 | ITLN1 | 0.0093 |
| 46 | KLHL4 | 0.011837 |
| 47 | LAIR1 | 0.040338 |
| 48 | LILRB2 | 0.001027 |
| 49 | MAFB | 0.012352 |
| 50 | MS4A4A | 0.032278 |
| 51 | MS4A6A | 0.019324 |
| 52 | MSR1 | 0.014131 |
| 53 | PDCD1LG2 | 0.048836 |
| 54 | PIK3AP1 | 0.00102 |
| 55 | PKHD1L1 | 0.004887 |
| 56 | PPARGC1A | 0.00061 |
| 57 | PTPRC | 0.029117 |
| 58 | RGS1 | 0.010007 |
| 59 | RNASE6 | 0.01294 |
| 60 | SIGLEC7 | 0.008771 |
| 61 | SLA | 0.043281 |
| 62 | SLCO2B1 | 0.017449 |
| 63 | TAT | 0.020108 |
| 64 | TMED6 | 0.020042 |
| 65 | WDR72 | 0.024467 |
| 66 | ZNF831 | 0.021313 |
